# Recent advances in understanding the role of HES6 in cancers

**DOI:** 10.7150/thno.72966

**Published:** 2022-05-20

**Authors:** Imène Krossa, Thomas Strub, Arnaud Martel, Sacha Nahon-Esteve, Sandra Lassalle, Paul Hofman, Stéphanie Baillif, Robert Ballotti, Corine Bertolotto

**Affiliations:** 1 Université Côte d'Azur, France; 2 Inserm, Biology and Pathologies of melanocytes, team1, Equipe labellisée Ligue 2020 and Equipe labellisée ARC 2019, Centre Méditerranéen de Médecine Moléculaire, Nice, France; 3 Centre Hospitalier Universitaire de Nice, Department of Ophthalmology, Nice, France; 4 Laboratoire de Pathologie clinique et expérimentale, biobanque BB-0033-00025, and IRCAN team 4, FHU OncoAge, Nice, France

**Keywords:** HES6, cancers, uveal melanoma, NOTCH, signaling

## Abstract

The NOTCH signaling system regulates a variety of cellular processes during embryonic development and homeostasis maintenance in different tissues and contexts. Hence, dysregulation of NOTCH signaling is associated with a plethora of human cancers, and there have been multiple efforts to target key components of this pathway. In this review, we briefly highlight the latest research advances in understanding HES6, a poorly studied component of the NOTCH pathway. We summarize the role of HES6 in cancers with a focus on uveal melanoma. Finally, we discuss the ongoing efforts to target the NOTCH-HES6 axis in cancers.

## Introduction

The NOTCH pathway remains a central focus in both basic and translational research.

Its activity is essential for normal embryonic development and tissue homeostasis. Dysregulated NOTCH signaling is associated with a variety of nonmalignant and malignant pathologies. To date, however, relatively little is known about the regulation of the NOTCH signaling pathway and target gene expression in various cellular contexts 1. Regarding cancer, while it is oncogenic in acute lymphoblastic leukemia 2, head and neck carcinoma 3 and cervical cancer 4, NOTCH signaling functions as a tumor suppressor in the development of skin carcinoma 5,6, hepatocellular carcinoma 7, urothelial carcinoma 8 and low-grade gliomas 9. How NOTCH mechanistically achieves tumor suppressive or promoter functions remains to be determined in most cancers. Therefore, better knowledge of upstream regulators or downstream NOTCH effectors in various cellular contexts is of paramount importance to design efficient therapeutic options based on the regulation of this signaling pathway.

**Figure 1:**
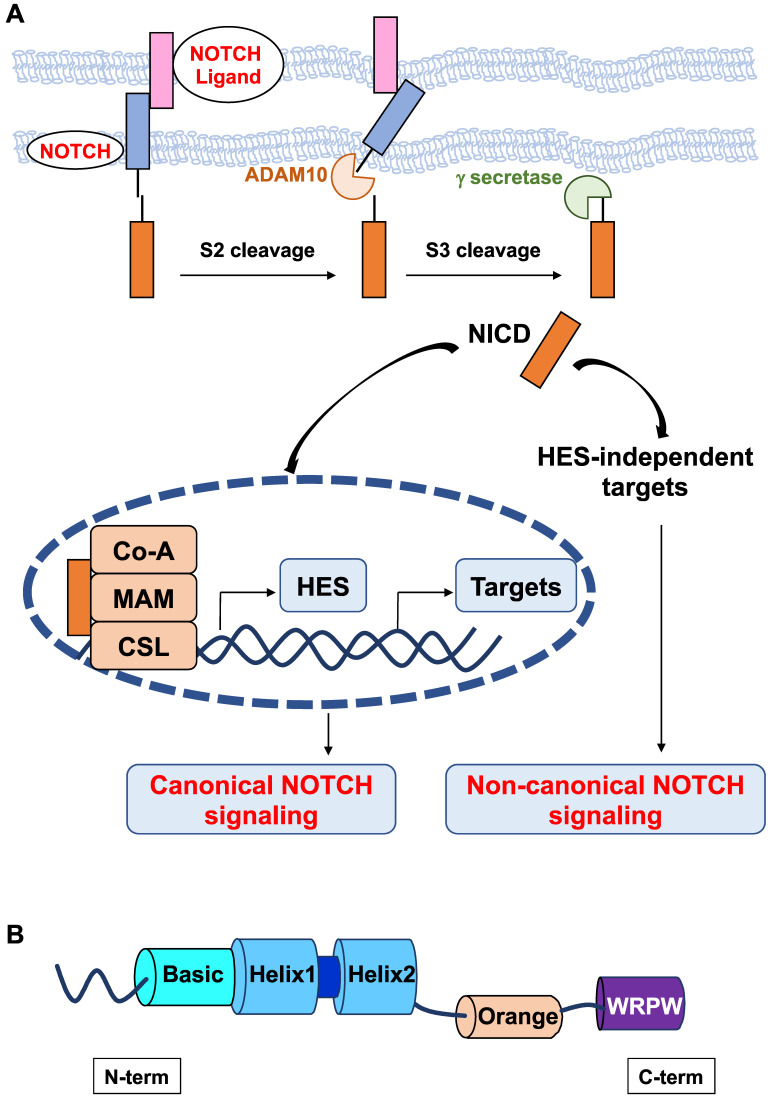
** Schematic representation of the canonical and noncanonical NOTCH signaling pathways. A.** The canonical Notch signaling pathway is activated by ligand-receptor interactions leading to NICD release and translocation to the nucleus to form a transcriptional activation complex after binding to MAML-1 and CSL. Noncanonical NOTCH signaling regulates target gene expression via mechanisms that do not implicate HES factors**. B.** Structure of HES factors with conserved domains.

In mammals, NOTCH receptors (NOTCH1-4) are activated following ligand binding (Delta-like DLL1, DLL3, DLL4 or Jagged JAG1, and JAG2). These ligands contain a DSL (Delta/Serrate/Lag2) domain that is required to interact with NOTCH. NOTCH activation is mediated by successive proteolytic cleavages; the latter involves γ-secretase, which leads to the release of the NOTCH intracellular domain (NICD) 10. NICD translocates to the nucleus and forms a ternary complex with the DNA-binding CSL (CBF-1/RBPJ-κ in mammals, Suppressor of Hairless in *Drosophila melanogaster*, Lag-1 in *Caenorhabditis elegans*) transcription factor and the transcriptional coactivator Mastermind Like transcriptional coactivator 1 (*MAML1*) to transactivate target genes (Figure 1A). NOTCH target genes include members of the hairy and enhancer of split (HES) family and HES-related with YRPW motif (HEY), which in turn regulate the transcription of downstream targets, such as proneural genes 11,12. There are seven members in the human HES (HES 1-7) family and three members in the human HEY (HEY1,2, L) family. From Drosophila to humans, the *HES* and* HEY* genes encode nuclear transcription factors that play a pivotal role in the development of many organs 13,14. In Drosophila, *Hes1, Hes5* and *Hes7* were found to be direct effector genes of the Notch signaling pathway 15, whereas* Hes2, Hes3* 16 and *Hes6* 17 appear to be independent of Notch signaling. Data on *Hes4* are lacking.

All HES proteins have three evolutionarily conserved domains, the basic helix loop helix (bHLH), Orange, and WRPW domains (Figure 1B). In the bHLH domain, the basic region, which is mainly composed of basic residues, is responsible for DNA binding, and the helix-loop-helix region, primarily comprising hydrophobic residues, allows these proteins to dimerize. The Orange domain regulates the specificity of bHLH dimer partners, and the C-terminal WRPW domain is implicated in transcriptional repression 18. The conserved WRPW motif of HES factors mediates their proteasomal degradation 19. As such, HES factors have very short half-lives (~20 minutes) 20. Despite their structural similarity, Hes-related factors have been classified into three subgroups according to the sequence homology in the bHLH and Orange domains: the Hes1-4 subgroup, Hes5/7 subgroup, and Hes6 subgroup 21. Instead of a WRPW tetrapeptide, a related YRPW peptide (HEY1,2) or a further degenerated YXXW (HEYL) are found in HES-related factors HEY.

bHLH factors usually bind to a hexanucleotide consensus sequence called the E box (CANNTG) that is present in the promoter region of their target genes. Although they belong to the bHLH family of transcription factors, some HES proteins have been reported to bind canonical E boxes (CANNTG) but with low affinity only and to bind to a different target sequence, namely, the N box (CACNAG) with high affinity 22,23 or they appeared not to bind DNA 22,24.

HES factors can exert transcriptional repression through active and passive mechanisms 25. Indeed, they can function as homodimers or as heterodimers with other bHLH factors, such as HEY1 and HEY2, and recruit Groucho(Gro)/transducin-like Enhancer of split (TLE) transcriptional corepressors at specific DNA sites. HES factors can also perform passive repression by forming heterodimers with bHLH activators bound to their E box, thereby preventing their DNA binding.

While NOTCH activation can trigger its effects through the canonical pathway that involves the expression of Hes genes, noncanonical NOTCH signaling has also been reported (Figure 1). Noncanonical NOTCH signaling is RBP-Jκ-and Hes independent and has been shown to be important in several cellular processes, including oncogenesis 26-28. Therefore, the noncanonical axis likely contributes to the pleiotropic effects of NOTCH signaling.

In this review, we focus on the role, regulation and mechanisms of action of HES6, with a special focus on cancers including uveal melanomas.

## Differentiation and progenitor maintenance

Hes6 was isolated by the group of Kageyama 22. Several lines of evidence indicate that Hes6 behaves differently from the other members of this family. It has been demonstrated to regulate multiple cell fate decisions in neural development and in muscle. Hes6 promotes neuronal differentiation, in contrast to other *Hes* genes that are associated with the inhibition of neurogenesis 17,22,29,30. Indeed, exogenous Hes6 expression in undifferentiated cortical progenitor cells was sufficient to induce neuronal differentiation 31. Furthermore, knockdown of Xenopus Hes6 (Xhes6) prevented neural differentiation, which could be rescued by reintroduction of both wild-type Xhes6 and an Xhes6 mutant unable to bind DNA 24. Xhes6 has been reported to function through the inhibition of antineurogenic Xhairy proteins and by interaction with Groucho/TLE family proteins for the induction of neurons mediated by both neurogenin and NeuroD 24. In addition to promoting neurogenesis, Hes6 inhibits astrocyte differentiation 32. Both the pro-neuronal role and anti-gliogenic functions require Hes6 nuclear localization but do not depend on its ability to bind to DNA. Two short peptides, LNHLL and WRPW, present in Hes6 proteins appeared to be important for anti-gliogenic functions but not for neurogenic functions 32. Moreover, a correlation has been observed between the ability of Hes6 to prevent transcriptional repression mediated by Hes1 (and/or other functionally related family members) and its anti-gliogenic activity, while this mechanism was not viewed as critical for the neurogenic function of Hes6 32. Collectively, these observations indicated that Hes6 pro-neuronal and anti-gliogenic functions involved distinct regulatory mechanisms. Hes6 expression in developing neurons was induced by proneural bHLH proteins such as neurogenins (NEUROG1, 2, 3) but not by the Notch pathway 17, suggesting that in this context, Hes6 functions through a Notch-independent pathway. Hes6 expression has also been detected in differentiated hair cells of the inner ear, where it has been shown to be a downstream effector of Math1 (ATOH1), another bHLH gene that is critically required for hair cell differentiation 33. Although Hes1 and Hes5 play an anti-neurogenic role in hair cell differentiation, Qian et al. did not demonstrate that Hes6 triggered its effect through the inhibition of HES1/5 functions 33.

Moreover, Hes6 has been reported to be a direct target of the myogenic factors MyoD and Myf5 and to control myoblast fusion, a fundamental step in the differentiation of muscle in most organisms 34,35. The myogenic functions of Hes6 appeared to be mediated by protein-protein interactions and not through its DNA-binding activity 23. One of the Hes6 dimerization partners could be Hes1. Hes6 has been shown to inhibit Hes1 activity either by blocking its interaction with Gro/TLE or by inducing proteolytic degradation of Hes1, thereby preventing Hes1 ability to mediate transcriptional repression 17,22,31. Since Hes1 is a critical NOTCH effector, Hes6 has been considered a negative regulator of the Notch pathway.

In addition to myogenesis and neurogenesis, HES6 has been implicated in the homeostasis and function of other tissues where its role still remains elusive. For instance, overexpression of Hes6 in the developing retina promotes rod photoreceptor differentiation through Hes1 inhibition, suggesting that they are involved in the activation of canonical NOTCH signaling 22. HES6 has been described to be enriched in a cell subpopulation that composes the upper airway epithelium and contributes to mucociliary epithelial differentiation and function 36. Finally, HES6 marks goblet cell precursors and the early stage of goblet cell differentiation in human gastric tissue 37.

Despite structural similarity, Hes6 seems to exhibit unique features within the Hes family. These features might rely on a loop region that is shorter than four or five amino acid residues compared to the other Hes factors 22. Indeed, it has been shown that Hes6 does not bind to the N box or E box sequences by itself to promote neuronal differentiation but functions through inhibition of Hes1 transcriptional repressive activity 22. However, the insertion of five amino acid residues in the loop allows Hes6 to exert N box-dependent repression activity, whereas the removal of five amino acids in the Hes1 loop region prevents the N-box-dependent repression activity of Hes1. Thus, the loop region appears critical for the specific functions of Hes6 and Hes1. Why the Hes6-Hes1 complex did not repress transcription has to be determined since both carry the WRPW repression domain. One explanation could reside in the structural organization of the WRPW domains in the complex preventing their interaction with corepressors (Groucho homologs) 22. Moreover, Hes6 might sequester Groucho homologs and inhibit the repression activity of Hes1. Another mechanism could be related to the loop of Hes1, which is longer than that of Hes6, enabling Hes1 to interact with cofactors of transcriptional repression.

## HES6 in cancer

The clinical significance and biological role of HES6 in human cancers remain poorly elucidated. Nevertheless, it might play a broad role due to amplification of the genomic region where HES6 is located in several cancers 38. Increased expression of HES6 has been detected in various tumors, including advanced astrocytoma, glioblastoma, prostate cancer, leukemia, gastric cancer, colon cancer, breast cancer, lung cancer, and kidney cancer and is associated with poor survival 37-41. It is worth noting that across different tumor types, *HES6* showed the highest enrichment in gliomas, suggesting that it might represent a lineage-specific cancer driver 42. Studies in colorectal cancers and glioma showed that HES6 represented a valuable prognostic biomarker 42-44. In the same vein, single-cell analysis of human premalignant gastric biopsies indicated that HES6 might mark the pregoblet cell cluster 37. The appearance of goblet cells in the human gastric epithelium, which is normally devoid of goblet cells, is a marker of possible malignant progression toward adenocarcinoma. Thus, HES6 potentially aids in the identification of metaplasia at the early stage 37.

Moreover, functional studies have revealed an important role for HES6 in supporting the growth and motile ability of cancer cells. HES6 increased proliferation in MCF-7 breast cancer cells *in vitro,* and this was confirmed in tumor xenografts *in vivo* 41. Moreover, enhanced HES6 expression stimulated the motile ability and invasive phenotype of prostate cancer cells, glioma cells and colorectal cancer cells 40,42,45. Conversely, HES6 knockdown decreased the migration of glioma, glioblastoma, alveolar rhabdomyosarcoma and colorectal cancer cells 42,43,45. The reduced motile ability of these tumor cells following HES6 knockdown may in part result from an interruption of NOTCH signaling. Altogether, these studies indicate that HES6 might be a valid therapeutic target. In progressive glioblastoma, inhibition of NOTCH signaling associated with an increase in HES6, which is known to repress the NOTCH effector HES1, was observed 46. Inhibition of NOTCH signaling was likely mediated by increased Delta1, which is able to interrupt NOTCH signaling 46. Other lines of evidence in favor of NOTCH signaling inhibition in this condition were the increased ASCL1 (Achaete-scute complex-like 1) level, which has been reported to be transcriptionally regulated by Delta1 expression and repressed by Hes1 46. It is worth noting that, compared to progressive lesions, in most primary glioblastomas, active NOTCH signaling was associated with low ASCL1 levels 46. Moreover, in glioma cells, HES1 was found to bind to a panel of HES6-regulated genes, and HES1 expression was reduced after HES6 downregulation 42. Thus, depending on the tissue context, HES6 can positively or negatively regulate HES1 effects.

HES6 has been recently implicated in uveal melanoma progression, which is the main primary intraocular malignancy in adults and the second most common melanoma. Despite successful treatment of primary uveal melanoma by radiotherapy or enucleation, up to 50% of patients will develop metastases, predominantly in the liver 47,48. Metastatic uveal melanomas are highly refractory to existing treatments. The only treatment that has been shown to improve the overall survival of patients is tebentafusp, a bispecific protein immunotherapy targeting CD3 and the melanoma antigen GP100 49. However, tebentafusp only works for patients with an HLA-A2 haplotype (40% of the cases) and is efficient in only 20% of them. Hence, ninety percent of patients will die within 6 months after the diagnosis of their metastases. Therefore, there is an urgent need to better understand the molecular mechanisms underlying the development and progression of uveal melanoma and to identify efficient therapeutic strategies.

The main known oncogenic drivers in uveal melanomas are mutations in the heterotrimeric G-protein alpha subunit GNAQ or its paralog GNA11 (GNAQ/11). Eighty percent of uveal melanomas harbor a mutation in one of these two genes. Mechanistically, oncogenic GNAQ/11 promotes ARF6 activation, which orchestrates the activation of a broad range of events, including the activation of the PKC/ERK and HIPPO/YAP signaling pathways to control the proliferation of uveal melanoma cells 50. GNAQ/11 driver mutations are coupled to coordinated events; the most frequent are the loss of the deubiquitinase *BAP1* and recurring chromosomal aberrations, such as chromosome 3 loss or 8q amplification, both associated with a high metastatic risk and a poor prognosis 51.

**Figure 2:**
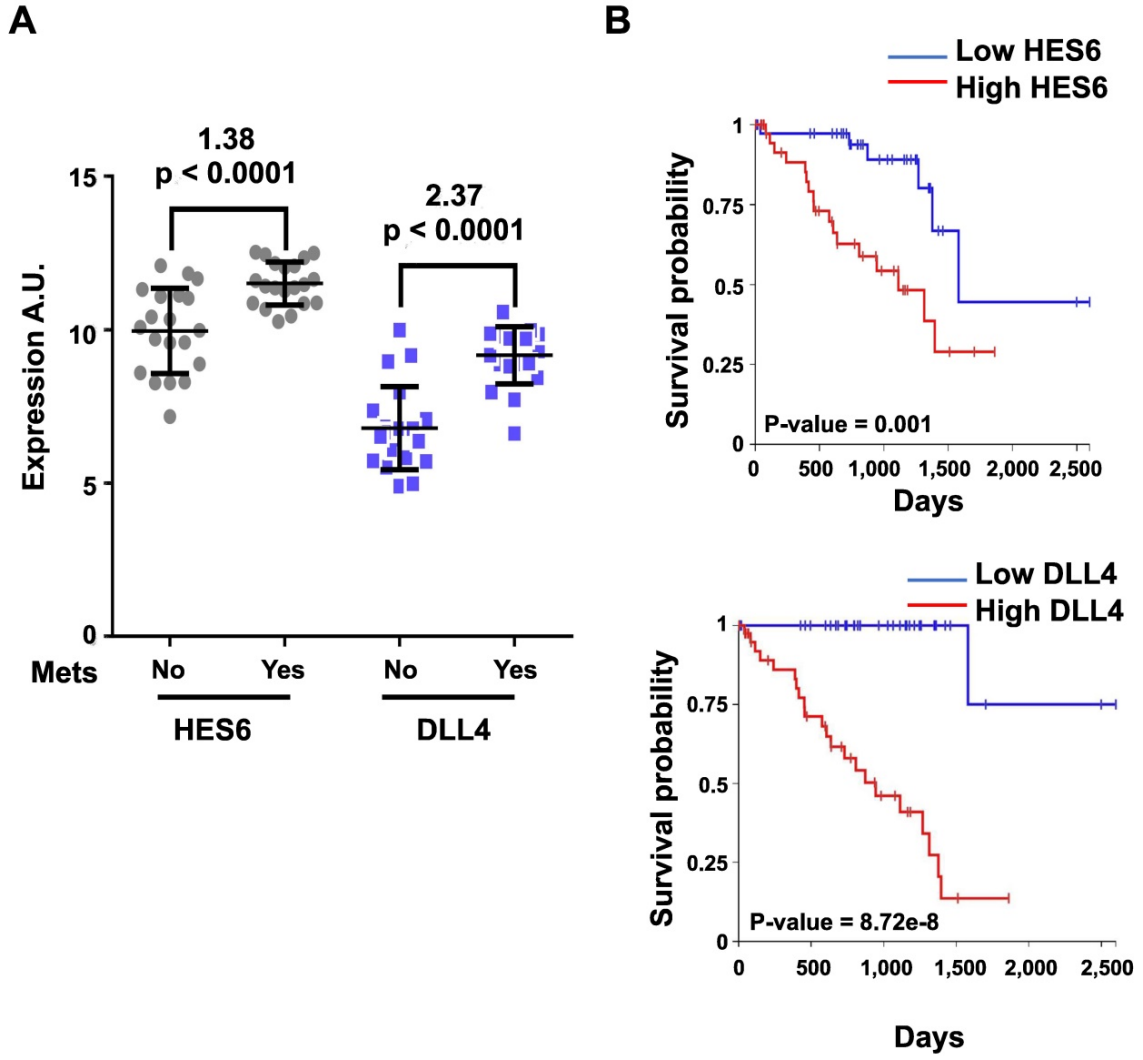
** HES6 and DLL4 are associated with metastasis formation and a poor prognosis.** The y-axis shows the log(2)-expression in patients who did not develop metastasis after a 2-year follow-up versus those who developed metastasis (Mets) before 18 months since UM diagnosis (Mets: No/Yes). Values indicated at the top of the figure correspond to log(2)-fold changes and p values of metastatic versus nonmetastatic patients. **B.** Kaplan-Meier curves showing overall survival stratified by HES6 and DLL4 expression (UM-TCGA dataset).

**Figure 3:**
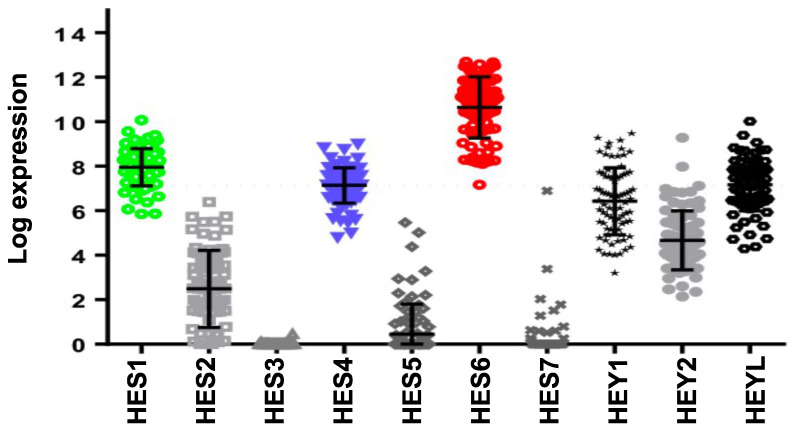
** Expression of HES/HEY members and correlation with prognosis of the NOTCH family.** Relative expression of HES/HEY gene family (UM-TCGA dataset).

Activation of NOTCH signaling was reported to play a key role downstream of oncogenic GNAQ in uveal melanoma cells. MRK003, a NOTCH signaling inhibitor, reduced the viability and migration induced by mutant GNAQ 52. Recently, single-cell RNA-seq analysis of freshly enucleated primary uveal melanomas revealed intratumor heterogeneity, which is considered a source of metastatic dissemination and therapy resistance in cancers 53-55. Interestingly, an invasive cell state driven, at least in part, by HES6 was identified 55,56. Consistent with this finding, HES6 expression was strongly associated with metastatic risk (UM-TCGA dataset). Thus, HES6 appears to be a relevant prognostic biomarker in primary uveal melanomas 55. Supporting this notion is the recent identification of a four-gene signature of prognostic significance in uveal melanoma that includes HES6 57. Additional evidence for the role of *HES6* as a prognostic biomarker has been shown in primary cutaneous melanomas 58. HES6 also emerged as a therapeutic target in uveal melanoma cells. Indeed, its knockdown reduced their growth and motile ability both *in vitro* and *in vivo* 55. Furthermore, HES6 knockdown inhibited uveal melanoma cell migration induced by DLL4, one of the five NOTCH ligands. Hence, HES6 appeared to mediate the canonical NOTCH effect in uveal melanoma, yet HES6 targets responsible for these effects have not been explored 55. In agreement with this finding, DLL4 and HES6 expression were both strongly associated with metastasis formation (Figure 2A) and a poor prognosis in uveal melanomas (Figure 2B). It is worth noting that primary uveal melanomas also display significant expression of HES1 and HES4 as well as of HEY1-2 and HEYL (Figure 3). While the expression of HEY factors is not significantly associated with prognosis in primary uveal melanomas, that of HES1 and HES4 is (UM-TCGA dataset, not shown). JAG2, another NOTCH ligand whose effect may involve HES1 and HEY1, is also critically required for uveal melanoma cell proliferation and motile ability 59,60. Altogether, these data indicate a key role for NOTCH activation and HES6 in uveal melanoma progression.

In summary, much work remains to be done to precisely determine which and how HES/HEY factors are implicated in cancer. HES6 contributes to the metastatic phenotype in tumors, including uveal melanomas, and represents a potentially important avenue of research toward uncovering therapeutic targets.

## Mechanisms of HES6 action (Figure 4)

Transcriptomic analysis of glioma cells in which the expression of HES6 was reduced or enhanced revealed that the commonly dysregulated genes were associated with biological functions such as migration, invasion, cell-to-cell signaling and cell proliferation 42. These analyses highlighted an enrichment in MYC motifs in HES6-deregulated genes, indicating that HES6 can influence *MYC* expression or activity 42. NOTCH1 has also been reported to regulate MYC levels in leukemic cells 61,62. Thus, one could envision that in glioma cells, NOTCH1 mediates its effect through HES6 regulation, although this remains to be determined. On the other hand, MYC has been reported to bind the *HES6* gene promoter in the lymphoblastic leukemia HPB-ALL human cell line 63, and in aggressive human prostate cancer cells, HES6 expression is controlled by c-Myc 40. Collectively, these data suggest the existence of a regulatory loop between the NOTCH-HES6 module and MYC.

MYC is of particular interest in uveal melanomas given its localization on chromosome 8, whose amplification (8q) is one of the most common genetic abnormalities in this disease and is strongly associated with metastatic risk 64. HES6 has also been shown to be regulated by hypoxia-inducible factor (HIF1a) 65, which mediates the hypoxic response, a central hallmark of cancer progression and dissemination 66. It is worth noting that HIF-1a and MYC may bind directly to many of the same promoters 67. Altogether, these data suggest the existence of a network involving HES6, MYC and HIF-1a that may fuel tumor progression.

Interestingly, in colorectal cancer, NOTCH activation by DLL4 triggers the induction, through DAB1 and ABL, of the TRIO-RHO module to drive invasion and metastasis 68. In uveal melanoma cells, the TRIO-RHO/RAC signaling axis lies downstream of oncogenic GNAQ/11 and stimulates YAP, a critical component of the Hippo signaling pathway, which in turn controls cell proliferation 50,69. Additionally, the ADP-ribosylation factor (ARF)6 GTPase binds to TRIO and functions as a node orchestrating the activation of Rac1 and β-catenin to mediate the motile ability of uveal melanoma cells 50.

The WNT/β-catenin signaling pathway, which can promote cancer metastasis 70, has been described to mediate the protumorigenic effects of HES6. In colorectal cancer cells, HES6 overexpression led to the nuclear accumulation of β-catenin and changes in the expression levels of several WNT target genes, such as TCF1 and SLUG 43. Knockdown of TCF4, a central effector of the WNT/β-catenin pathway, prevented the migration and invasion of human colon cancer cells 70. c-MYC is also among the Wnt/β-catenin targets 71. In uveal melanomas, Wnt/β-catenin is overexpressed compared to normal melanocytes and is associated with decreased overall survival 72. Thus, MYC upregulation can result from 8q amplification or from activation of the Wnt/β-catenin signaling cascade. Wnt/β-catenin inhibition with antimalarial drugs 73,74 or ICG-001, an inhibitor of β-catenin/TCF-induced transcription, impaired uveal melanoma cell growth and induced apoptosis 75. Of interest, the drug caused suppression of MYC 75. Whether Wnt/β-catenin indeed lies downstream of HES6 in uveal melanoma cells and whether Wnt/β-catenin inhibitors prevent HES6 effects on proliferation and migration or MYC expression remain to be investigated. It is worth noting that activation of the β-catenin signaling pathway is known to cause immune evasion in cutaneous melanomas 76,77. Hence, HES6 might also contribute to tumor progression by preventing efficient antitumor immunity.

SLUG is a member of the Snail transcription factor family involved in epithelial-mesenchymal transition (EMT), a molecular process by which epithelial cells lose their cell-cell adhesion and gain migratory and invasive capabilities to favor malignant progression. The prototype of the EMT switch is the loss of CDH1, which encodes E-cadherin that mediates cell-cell adhesion. E-cadherin sequesters β-catenin on the cell membrane via its cytoplasmic tail. Consequently, its loss of function results in β-catenin release into the cytoplasm and translocation into the nucleus, where it triggers the expression of EMT-inducing transcription factors that are implicated in cancer and metastasis 78. Since HES6 can control both β-catenin and SLUG, HES6 may favor EMT, whereby cells lose their cell-cell adhesion and gain migratory and invasive properties. However, in uveal melanomas, CDH1 expression is maintained and is associated with a poor prognosis 79. Relevant to this, HES6 expression correlates with CDH1 (UM-TCGA, r=0.61, p value<0.001). Likewise, the NOTCH signaling pathway involves the interaction between two adjacent cells, one expressing a ligand (either Delta or Jagged) and the other expressing a NOTCH receptor. In this circumstance, uveal melanoma cells may not need to undergo a pseudo (melanoma cells are nonepithelial in nature)-EMT for progression. Supporting this idea, HES6 expression was more elevated in uveal melanoma tumors that predominantly contained epithelioid cells, a marker of poor prognosis 56. Therefore, uveal melanoma cells might remain mechanically connected and exhibit a collective form of migration to disseminate. Another important gene downstream of HES6 is NESTIN, a marker for cancer stem cells 42. In glioma cells, NESTIN is one of the E-box-containing genes that increased after HES6 overexpression. Interestingly, NESTIN has been described as a possible biomarker for high-risk uveal melanoma 80. Whether HES6 favors a pseudo-EMT and a stem cell phenotype in uveal melanoma cells remains to be determined.

## Therapeutic opportunities

Several lines of evidence indicate that NOTCH has a role in promoting resistance to both conventional chemotherapy 81-84 and targeted therapy 85,86, including in cutaneous melanomas 87,88. This may be due to “stemness or tumor dormancy induction. Several molecules targeting NOTCH signaling are currently in clinical development. However, since NOTCH signaling plays a key role in tissue homeostasis and in the antitumor immune response, the clinical development of these therapies is more complex than expected 89.

Gamma secretase inhibitors (GSIs) have been reported to induce cancer stem-like cell differentiation and apoptosis, to impair EMT and to sensitize cells to traditional chemoradiotherapies *in vitro* and in preclinical models. For instance, BMS-906024 enhanced the anticancer efficacy of paclitaxel compared to either drug alone in lung adenocarcinoma 90. In preclinical models of uveal melanoma, MRK003 prevented tumor growth and metastatic dissemination 60. NOTCH inhibitors are also capable of sensitizing cancer cells to radiation 91. Given that proton beam radiation is the “gold standard” treatment for primary uveal melanomas, the use of NOTCH inhibitors appears highly relevant in this disease context. The manner by which NOTCH blockade affects tumor growth is likely through inhibition of AKT, ERK and STAT3 activity, as demonstrated in uveal melanoma cells 60.

To date, NOTCH inhibitors alone or combined with other therapies have failed to translate into beneficial effects in most solid tumors, with the notable exceptions of central nervous system malignancies and desmoid tumors 92. Phase III clinical trials of Rova‑T for patients with small‑cell lung cancer and of nirogacestat for patients with desmoid tumors are ongoing 93. One important obstacle to using NOTCH as a therapeutic target is that its signaling pathway can trigger oncogenic or tumor‑suppressive effects in a cancer stage‑ or (sub)type‑dependent manner. How NOTCH achieves these distinct functions awaits further investigation. Furthermore, NOTCH has broad functions and controls the fate of various T-cell types and myeloid cells that may favor tumor-supporting immunogenicity, helping the tumor evade the immune response. Thus, strategies could be a dosage de-escalation of anti-NOTCH therapies or combination with different therapies as well as designing specific treatment schedules. The lack of substrate specificity of NOTCH inhibitors and the associated toxicity constitute significant limitations to their therapeutic use. Tumor-targeted drug delivery might help exclude the systemic side effects of anti-NOTCH drugs.

NOTCH also plays an essential role in coordinating vessel development and maintenance 94 and cancer-related neoangiogenesis, in which the latter is mediated through the ligand DLL4. In human tumors, DLL4 expression was thought to be restricted to the tumor vasculature, and its expression appeared to correlate with the clinical outcome in a panel of tumors, such as breast, ovarian, gastric and resected pancreatic tumors 95-98. Of interest, among the NOTCH ligands, DLL4 is highly expressed in uveal melanoma cells, and it is the member whose expression is the most strongly associated with their metastatic ability 56. Consequently, DLL4 inhibition in uveal melanomas would be a logical approach since they have a strong propensity to metastasize via the hematogenous route. DLL4 blockade has measurable antitumor effects in animal models of various cancers 99,100. Therefore, DLL4 has also emerged as an attractive target for antiangiogenic cancer therapy and provides an additional level of control for blocking tumor growth. However, therapeutic antibodies targeting DLL4 (demcizumab), which are expected to exhibit better efficacy and specificity than small molecule inhibitors, have not met the expected endpoints 101.

Another possibility would be targeting downstream NOTCH effectors. As previously indicated, HES6 expression is increased and associated with a poor prognosis in some cancers. Its detection may help to assess a patient's risk of disease progression, thereby allowing better follow-up and improving clinical decision-making. In the context of uveal melanoma, HES6 detection may also serve patient stratification for administration of adjuvant therapy rendered possible with the recent discovery of tebentafusp 49.

**Figure 5:**
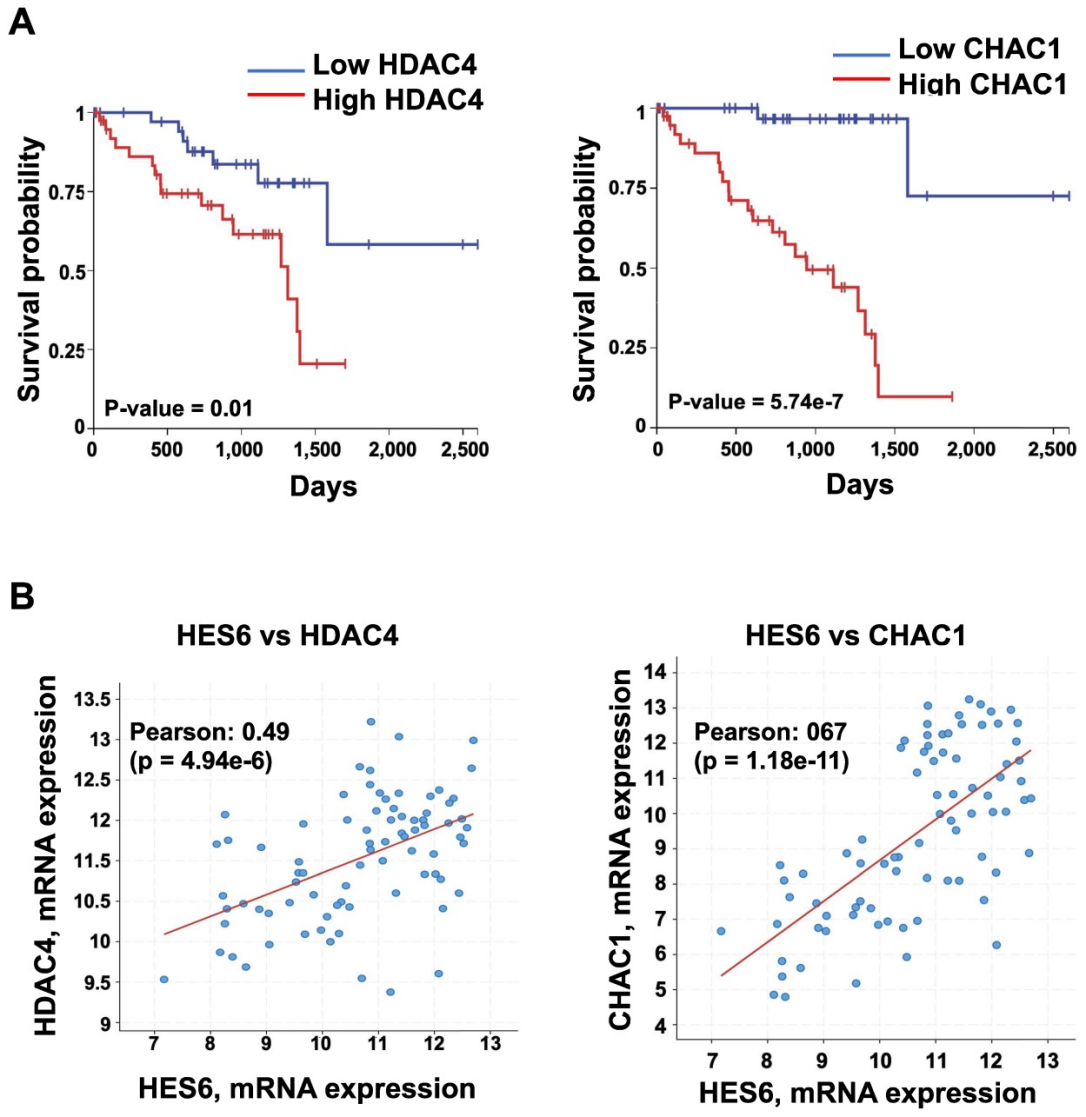
** Expression of HDAC4 and CHAC1 and their correlation with prognosis. A.** Kaplan-Meier curves showing overall survival stratified by HDAC4 and CHAC1 expression (UM-TCGA dataset). **B.** Expression correlation between HES6 and HDAC4 or CHAC1 (https://www.cbioportal.org).

HES6 represents a potentially important avenue of research toward uncovering therapeutic targets. Currently, there are no available direct HES6 inhibitors. Therefore, understanding how HES6 is regulated and functions remains a logical goal to identify potential upstream regulators and/or downstream effectors that may be targeted. Our single-cell analysis of primary uveal melanomas identified a list of HES6 effectors (HES6 regulon). These may represent therapeutic targets in uveal melanomas but also in other tumor types. Among them, histone deacetylase 4 (*HDAC4*) and ChaC glutathione-specific γ-glutamylcyclotransferase 1 (*CHAC1*) expression correlated with poor prognosis (UM-TCGA dataset) (Figure 5A-B). Inhibition of either HDAC4 or CHAC1 reduced the proliferation of uveal melanoma cells 102,103. CHAC1 is an effector of the endoplasmic reticulum stress pathway that has been shown to promote chemotherapy resistance in uveal melanoma cells 104. Hence, CHAC1 inhibition might offer therapeutic benefit for cancer treatment. Other avenues for exploration with growing interest in therapy include proteolysis targeting chimeras (PROTAC) technology. PROTAC utilizes the ubiquitin‐protease system to target a specific protein and induce its degradation in the cell 105. Importantly, studies have shown that protein degradation provides a superior effect than its inhibition of anticancer activities 106. Thus, targeting HES6 through the PROTAC approach may represent a potential therapeutic strategy for the treatment of cancers, including uveal melanomas. Finally, one might envision applying a network pharmacology strategy to simultaneously target HES6 and/or other NOTCH-related targets, which may produce therapeutic synergistic effects and translate into better therapeutic efficacy in different cancers.

## Conclusion

Patients with uveal melanoma are in therapeutic need. We anticipate that information gained from basic research will lead to a paradigm shift in the management and outcomes of uveal melanomas by identifying new druggable frailties to limit metastatic dissemination and metastatic growth. HES6 appears to be a valuable prognostic biomarker that could enable patient stratification for adjuvant treatment, and it may be a valuable therapeutic target in different cancers.

## Figures and Tables

**Figure 4:**
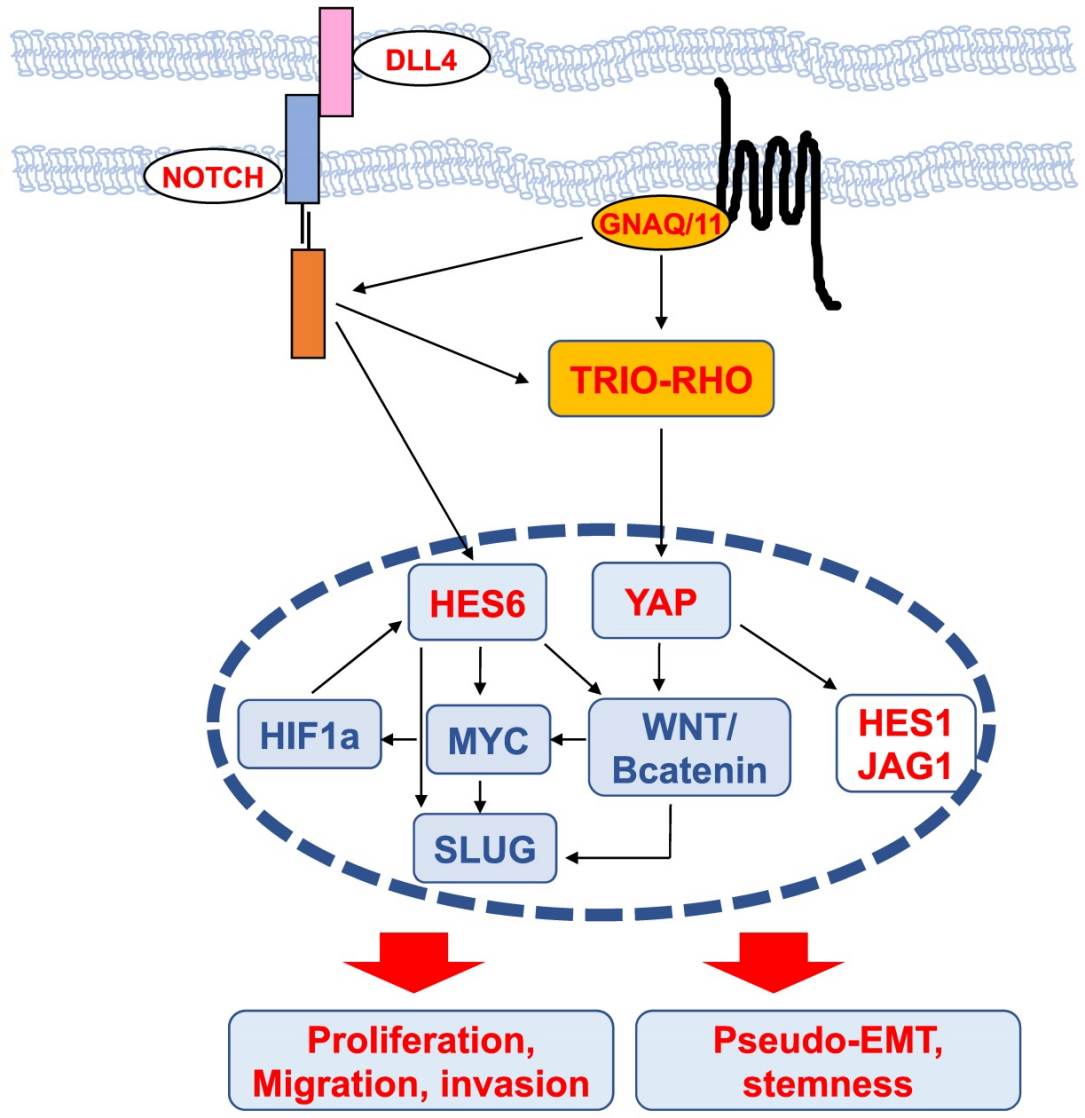
** Scheme of upstream regulators and downstream effectors of HES6 described in different cancers.** The nodes identified in uveal melanomas are shown in red.
